# Aβ-CT Affective Touch: Touch Pleasantness Ratings for Gentle Stroking and Deep Pressure Exhibit Dependence on A-Fibers

**DOI:** 10.1523/ENEURO.0504-22.2023

**Published:** 2023-05-23

**Authors:** Laura K. Case, Nicholas Madian, Micaela V. McCall, Megan L. Bradson, Jaquette Liljencrantz, Benjamin Goldstein, Vincent J. Alasha, Marisa S. Zimmerman

**Affiliations:** 1 Department of Anesthesiology, University of California San Diego School of Medicine, San Diego, CA 92037; 2 National Center for Complementary and Integrative Health, National Institutes of Health, Bethesda, MD 20892; 3 Department of Anesthesiology and Intensive Care, Institute of Clinical Sciences, Sahlgrenska Academy at University of Gothenburg, 413 45 Gothenburg, Sweden

**Keywords:** affective touch, C-tactile, pleasant touch, sensory afferents

## Abstract

Gentle stroking of the skin is a common social touch behavior with positive affective consequences. A preference for slow versus fast stroking of hairy skin has been closely linked to the firing of unmyelinated C-tactile (CT) somatosensory afferents. Because the firing of CT afferents strongly correlates with touch pleasantness, the CT pathway has been considered a social-affective sensory pathway. Recently, ablation of the spinothalamic pathway- thought to convey all C-fiber sensations- in patients with cancer pain impaired pain, temperature, and itch, but not ratings of pleasant touch. This suggested integration of afferent A and CT fiber input in the spinal cord, or mechanoreceptive A-fiber contributions to computations of touch pleasantness in the brain. However, contribution of mechanoreceptive A-fibers to touch pleasantness, in humans without pain, remains unknown. In the current, single-blinded study, we performed two types of peripheral nerve blocks in healthy adults to temporarily eliminate the contribution of A-fibers to touch perception. Our findings show that when mechanoreceptive A-fiber function is greatly diminished, the perceived intensity and pleasantness of both gentle stroking and deep pressure are nearly abolished. These findings demonstrate that explicit perception of the pleasantness of CT-targeted brushing and pressure both critically depend on afferent A-fibers.

## Significance Statement

In the current study we performed two types of peripheral nerve blocks in healthy adults to temporarily eliminate the contribution of A-fiber afferents to touch perception. We show that when afferent A-fiber function is greatly diminished, the perceived intensity and pleasantness of gentle stroking are nearly abolished. These findings demonstrate for the first time that explicit perception of the pleasantness of C-tactile (CT)-targeted touch critically depends on A-fiber afferents. In addition, we show the same outcome for deep pressure (similar to hugs and massage), another form of social-affective touch we have previously validated in the lab. Together these findings demonstrate that social touch is not conveyed solely by the CT pathway.

## Introduction

While top-down effects of mood and social context strongly shape the affective nature of touch (Sailer and Leknes, 2022), there is evidence that bottom-up sensory afferents prime the affective valence of pleasant touch, much as stimulation of nociceptors frequently leads to pain. Conventionally, myelinated Aα and Aβ afferents convey proprioceptive and touch signals, while thinly myelinated Aδ and unmyelinated C-fibers relay temperature, chemical, and pain signals (Burgess and Perl, 1967). However, the pleasantness of gentle stroking has been linked to a subset of C-fibers called C-tactile (CT) afferents, which are maximally activated by slow gentle stroking (Vallbo et al., 1999). The firing of CT fibers correlates with ratings of the pleasantness of gentle stroking (Löken et al., 2009), and CT touch activates the posterior insula (Olausson et al., 2002) and increases positive affect (Pawling et al., 2017). Given their affective effects and anatomic distinction from the Aβ pathway, it is argued that CT fibers subserve a distinct social-affective pathway described in the “Social Touch Hypothesis” (Vallbo et al., 1999; Morrison et al., 2010), while afferent Aβ fibers predominantly support discriminative aspects of touch (McGlone et al., 2007; Olausson et al., 2008; Morrison et al., 2010; Gordon et al., 2013).

Affirming the role of CT fibers in touch pleasantness, patients with hereditary reductions in C-fiber afference exhibit reduced preference for slow stroking (Morrison et al., 2011), while patients with A-fiber deafferentation report mild pleasantness of CT-targeted touch and show CT touch-induced insula response (Olausson et al., 2002, 2008). Patients with a functional loss of the *PIEZO2* ion channel subserving mechanotransduction, who exhibit severe tactile deficits, similarly remain able to detect CT-targeted slow stroking on hairy skin (Chesler et al., 2016). However, these results stem from small studies of patients with rare sensory abnormalities or disease, who may have abnormal sensory development or compensatory brain plasticity.

There is also evidence that pleasant touch perception may require convergent A- and C-fiber inputs. This was postulated early in the CT theory (summary published previously; Vallbo et al., 2009), and recent findings contribute positive evidence. First, in rodents, some Aβ and CT afferents converge onto common interneurons in the spinal cord (Abraira et al., 2017). Second, in humans with intractable unilateral cancer-related pain, ablation of the Lamina I-spinothalamic pathway, the putative pathway for all unmyelinated afferents, largely eliminates perception of pain, temperature, and itch, but does not eliminate the pleasantness of slow stroking (Marshall et al., 2019). Finally, electroencephalography (EEG) recordings demonstrate modulation of primary somatosensory cortex by gentle stroking temporally preceding the slower CT signal, and correlated with touch pleasantness ratings, suggesting CT modulation of dorsal column (Aβ-associated) spinal projections(Schirmer et al., 2022), while magnetoencephalography (MEG) recordings show activation of more affective brain areas such as the insula and cingulate by A-fibers during naturalistic stroking (Hagberg et al., 2019). Indeed, it has been hypothesized that stimulation of CT afferents might act as positive reinforcement for gentle tactile interaction in the social development of infants (Ackerley, 2022; Croy et al., 2022).

Furthermore, gentle stroking on the glabrous skin of the palm, where CT fibers are scarce (Watkins et al., 2021), still elicits (slightly lower) ratings of conscious pleasantness (Löken et al., 2011; Klöcker et al., 2012; Cruciani et al., 2021). Together with the aforementioned findings, this result suggests a potential critical role of Aβ mechanoreceptive afferents in pleasant touch perception- however, the contribution of the scarce CTs is not known. In sum, it is not known whether Aβ mechanoreceptive afferents are required for pleasant touch perception in the moment of touch, in healthy humans, or whether CT fibers can be sufficient.

In addition to the pleasant effects of gentle stroking, our research also confirms the pleasant and relaxing effects of deep pressure, as in massage (Case et al., 2021b). However, the mechanism for this sensation is not known. In humans, cutaneous anesthetic block eliminates skin sensation with little alteration in the sensation of deep pressure (Graven-Nielsen et al., 2004), suggesting distinct pathways for deeper pressure sensation. Indeed, nerve compression blocks first block cutaneous sensation, then deep pressure, and finally deep pressure pain (Kellgren and McGowan, 1948), and animal research has shown that both myelinated and unmyelinated sensory afferents in muscle can respond to pressure (Kaufman et al., 1983; Mense and Meyer, 1985; Abrahams, 1986; Lewin and McMahon, 1991). Consistent with these findings, our human research has shown the dependence of pressure intensity sensing on Aβ afferents, with a non-*Piezo2* mechanism for mechanotransduction (Case et al., 2021a). However, the neural mechanisms for pleasantness perception have not been studied.

Here, we conduct two types of temporary A-fiber blockades to determine the contribution of mechanoreceptive A-fiber afferents to conscious perception of the pleasantness of gentle stroking and deep pressure, at the time of touch. Ischemic nerve block (Study 1) yields clear separation of A- and C-fiber functions (Laursen et al., 1999) for a large area of skin, but causes pain and discomfort. Nerve compression block (Study 2) affects a smaller skin surface area, but with minimal discomfort- and has previously been used to correlate specific nerve afferents with sensory percepts (Wahren et al., 1989; Wasner et al., 2004; Forstenpointner et al., 2019). Furthermore, the latter technique has demonstrated preferential blockade of A-fibers during microneurography recordings in humans (Torebjörk and Hallin, 1973; Mackenzie et al., 1975). These nerve block techniques offer complementary strengths and weaknesses that together afford a robust test of the contribution of afferent A-fibers to affective qualities of touch, in the moment of touch.

## Materials and Methods

### Study 1

#### Participants

Study 1 was approved by the National Institutes of Health Intramural Institutional Review Board. This study was a preliminary study and no sample size calculation was performed. Healthy controls were selected based on age and sex from participants in a broad screening protocol at National Center for Complementary and Integrative Health (NCCIH). Potential participants were scheduled for a telephone screening during which the study procedures were described, and eligibility criteria were reviewed. Participants underwent medical screening and were excluded if they had unstable medical or psychiatric conditions and any abnormalities of the skin or nerves. All participants provided informed consent and were financially compensated for their time. A total of seven healthy volunteers participated; complete data with successful separation of A- and C-fiber nerve function was obtained and analyzed from five participants (two female and three male, ages 21–25).

#### Methods

##### Baseline affective touch task

**Figure 1.**
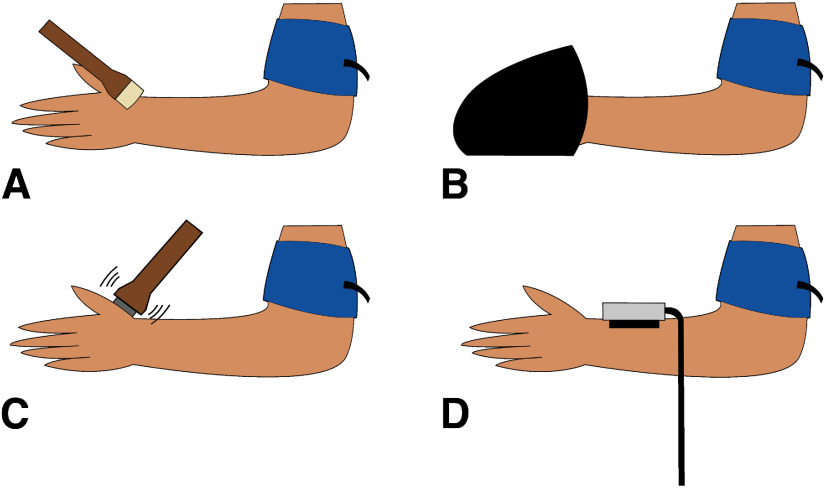
Somatosensory stimuli administered during ischemic compression nerve block. ***A***, Gentle brushing was administered with at a rate of 3 cm/s using a soft goat hair watercolor brush. ***B***, Deep pressure was administered using a commercially available hand massager. ***C***, Vibration sensation was tested using a custom vibration device at 200 Hz. ***D***, Perception of warmth was tested using a Medoc thermode.

At baseline each participant received gentle brushing (back of the hand at a rate of 3 cm/s for 15 s using a soft goat hair watercolor brush; Fig. 1*A*). Participants rated each of these stimuli on two visual analog scales, one for intensity [anchors of “no sensation” (coded as 0) to “highest possible intensity” (coded as 100)] and one for pleasant/unpleasantness [anchors “extremely unpleasant” (−100) to “neutral” (0) to “extremely pleasant” (100)]. Participants then received oscillating deep pressure from a commercially available hand massager (Daiwa Felicity – Acu Palm Hand Massager, Model No. USJ-881; Fig. 1*B*) for 20 s, and rated it on the same intensity and pleasant/unpleasantness scales. The massager had three preset patterns and each participant sampled them and selected the most pleasant to use at the beginning of the study. All patterns administered very deep pressure between the wrist and the hand, but force and frequency information was not provided by the manufacturer. Testing was conducted on the arm to be blocked and then on the control arm.

##### Nerve block placement

The participant’s left arm (this was the nondominant arm for three of five participants) was elevated above the head and exsanguinated for ∼1 min. Then, an automated blood pressure cuff device was wrapped around the brachium of the arm and was rapidly inflated to ∼100 mmHg above the participant’s systolic blood pressure. The arm was then rested on a pillow with the dorsal side down. Vital signs were monitored at regular intervals.

##### Nerve function monitoring

We started with four baseline rounds of testing, which included tests of several different sensory stimuli that have known associations with specific afferents. To track Aβ function we used a custom vibration device that applied 200-Hz vibration for a random interval of 1–6 s on a 1.3 × 4 cm region of skin on the lower palm near the wrist using a custom-built probe (4.0 × 1.2 × 0.7 cm of balsa wood connected to a piezo-element (Piezo Systems); previously used by Liljencrantz et al., 2017; Fig. 1*C*). Participants reported the onset and offset of vibration verbally over a set of three trials. To track C-fiber function we applied a Medoc thermode (Medoc; Fig. 1*D*) over the ventral forearm at 32°C, and increased the temperature at a rate of 1°C/s until the participants indicated perception of warmth by a button press. Additional somatosensory tasks for other purposes were conducted that are not reported here. The vibration and warmth threshold tasks were repeated approximately every 2 min until a substantial loss of vibration detection (<50% detection) was observed.

##### Final affective testing

The baseline affective touch task was repeated directly after loss of vibration perception.

During all testing the participants wore noise-isolating headphones playing white noise and had a visual barrier obscuring their vision of the stimuli.

##### Data analysis

Study 1 was a preliminary study with lower power. We conducted paired t tests to compare ratings of pleasantness and intensity before versus after nerve block.

### Study 2

We initiated Study 2 to overcome limitations of Study 1, particularly the painful and aversive nature of the ischemic nerve block. Study 2 was preregistered with the Open Science Framework, https://osf.io/q2b68.

#### Participants

Study 2 was approved by the University of California San Diego Biomedical Institutional Review Board. Given the Cohen’s *d* effect sizes of 1.3 and 1.6 in Study 1, and assuming a within-subject correlation of 0.5 and an attrition rate of 35%, a sample size of 24 was proposed to provide >0.8 power to detect an effect size of at least *d *=* *0.8 with a two-sided α = 0.05. Healthy controls were recruited from the local university and community, and from previous studies. Potential participants were scheduled for a telephone screening during which the study procedures were described, and eligibility criteria were reviewed. Participants were included if they were 18–50 years of age, right-handed, fluent in English, and had no indication of chronic pain or current pain. Participants were excluded if they had body mass index (BMI) >40, unstable psychiatric conditions, current opiate use or pregnancy (urine drug screen), current lactation, history of fainting from medical procedures, allergies to latex, major medical conditions, sensory or motor abnormalities, coagulopathy or use of anti-coagulant medications, inability to communicate with investigator or rate sensations, nerve block site infection or injury, or any other medical counterindications to nerve block. All participants provided informed consent and were financially compensated for their time. A total of 24 healthy volunteers participated (7 male and 17 female; ages 20–50; self-reported ethnicity five White, five Hispanic, six Asian, one mixed; M =* *26.8, SD =* *7.64). There was no overlap in participants between Studies 1 and 2.

#### Methods

Participants completed a urine pregnancy test and opiate drug test.

Perception of affective touch (Brushing rating task and Pressure rating task) was tested before and after the nerve block took effect, first on the blocked arm and then on the control arm. All testing was conducted within the region of the dorsal hand affected by the compression block.

##### Brushing rating task

**Figure 2.**
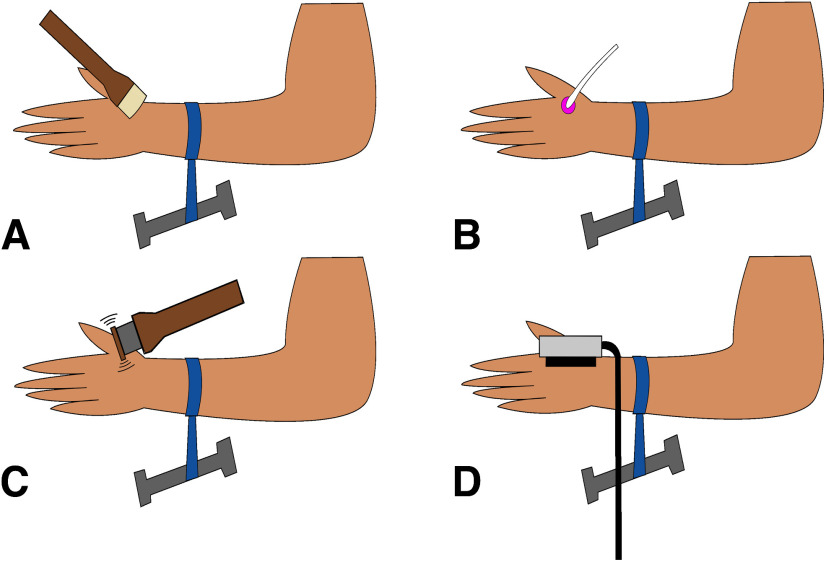
Somatosensory stimuli administered during nerve compression block. ***A***, Gentle brushing was administered with at a rate of 3 cm/s using a soft goat hair watercolor brush. ***B***, Deep pressure was administered using a commercially available hand massager. ***C***, Vibration sensation was tested using a custom vibration device at 200 Hz. ***D***, Perception of cold and warmth were tested using a QST.Lab T09 thermode.

First, gentle brushing (Fig. 2*A*) was administered sequentially to the blocked and control arm for 15 s each, using the side of a goat hair watercolor brush (<1 cm). At the end of each brushing period, participants made ratings on two visual analog scales of intensity [anchors of “no sensation” (later coded as 0) to “highest possible intensity” (100)] and pleasant/unpleasantness [anchors “extremely unpleasant” (later coded as −100) to “neutral” (0) to “extremely pleasant” (100)].

##### Pressure rating task

Deep pressure was administered to the blocked and control arm for 15 s each using a handheld rolling massage ball (Fig. 2*B*), applied by the experimenter to the dorsal area of the hand between the thumb and pointer finger. The massage ball was rolled across the area repeatedly in a proximal to distal direction at a slow velocity similar to the brushing velocity and an approximate force of 1–1.2 N. Participants rated intensity and pleasant/unpleasantness as in the Brushing rating task.

##### Baseline nerve function tasks

Cold detection, vibration detection, and warmth detection were assessed at baseline, before placement of the nerve block. Each task was comprised of three trials and the mean of the three trials was taken to establish baseline sensory function. The same vibration task was used as in Study 1, but vibration was applied to the dorsal hand (Fig. 2*C*). In the cold detection task, a QST.Lab T09 thermode (QST.Lab) was placed on the dorsal hand in the area anticipated to be blocked (Fig. 2*D*). The thermode started at the participant’s skin temperature and was lowered at a rate of 2°C/s until the participant indicated their perception of a cooling sensation via a response button. In the warmth detection task, the thermode was placed on the dorsal hand and increased at a rate of 2°C/s until the participant indicated their perception of a warming sensation.

The Brushing rating task, Pressure rating task, and Baseline nerve function tasks were each conducted a second time to provide familiarization and comfort with the tasks before nerve block placement.

##### Nerve block placement

We initiated a nerve compression block over the left superficial radial nerve following validated procedures (Ziegler et al., 1999; Nahra and Plaghki, 2003; Forstenpointner et al., 2019): while the left hand rested in semi-prone position, a ∼1-inch cloth tourniquet was placed over the left forearm ∼7 cm from the wrist. A five-pound weight was dangled from the tourniquet, similar to the weights used in some nerve compression studies (Wahren et al., 1989; Fig. 2). This technique often takes an hour to achieve loss of touch and cold perception (Nahra and Plaghki, 2003), but does not affect major blood vessels or induce significant pain (Wasner et al., 2004). The block was released within a common safety time window of 90 min for healthy research participants (Forstenpointner et al., 2019).

##### Nerve function monitoring

After block placement, cold and warm detection thresholds were monitored every ∼5 min following the same procedure as at baseline. The first two rounds of monitoring were used to establish baseline sensory nerve function. The function of Aβ fibers was monitored by the vibration task and cold threshold, with a loss of Aβ mechanoreceptive afferents function determined by vibration perception <50% (as in our previous study; Case et al., 2021a), and a drop in cold threshold of >5°C. The anesthetic zone was monitored with a cotton swab, given variability in distribution of the superficial radial nerve (Keplinger et al., 2018), and stimulus placement was adjusted accordingly. The continued function of C-fibers was confirmed by warm thresholds maintained within 1°C of baseline (Wahren et al., 1989).

##### Postblock affective touch testing

The Brushing ratings task and Pressure ratings task were repeated directly after the loss of vibration and cold detection.

##### Final nerve function confirmation

After the nerve block was achieved and the affective touch testing was completed, a final round of nerve function testing was conducted to confirm maintained loss of A-fiber sensation and preservation of C-fiber function.

Upon completion of all test procedures or upon reaching the 90-min safety limit, the tourniquet was removed, and sensory function was quickly restored to baseline.

##### Data analysis

In Study 2, we conducted linear mixed effect analyses using pleasantness and intensity as dependent measures, time, arm, and their interaction as fixed effects, and participant intercept and slopes as random effects.

## Results

### Study 1

**Figure 3.**
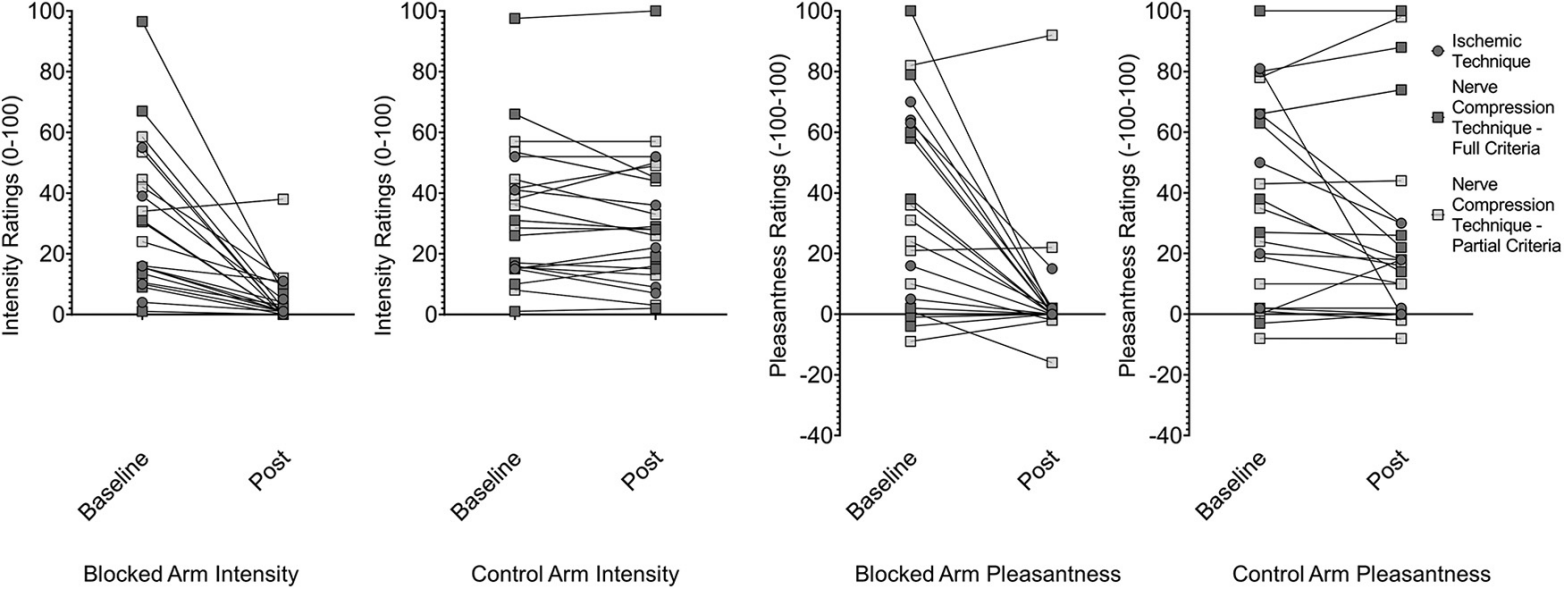
Effect of afferent A-fiber block on intensity and pleasantness of gentle brushing. The intensity and pleasantness of slow gentle brushing on the hand or arm was rated after ischemic or compression nerve block, on sufficient loss of A-fiber associated sensation. Participants who met all preestablished criteria for nerve fiber separation and maintained the criteria after affective testing are labeled “full responders”; participants whose warmth perception rose >1°C or who did not maintain all criteria directly after the brushing task are labeled “partial responders.” For pleasantness ratings, negative numbers indicate unpleasantness.

**Figure 4.**
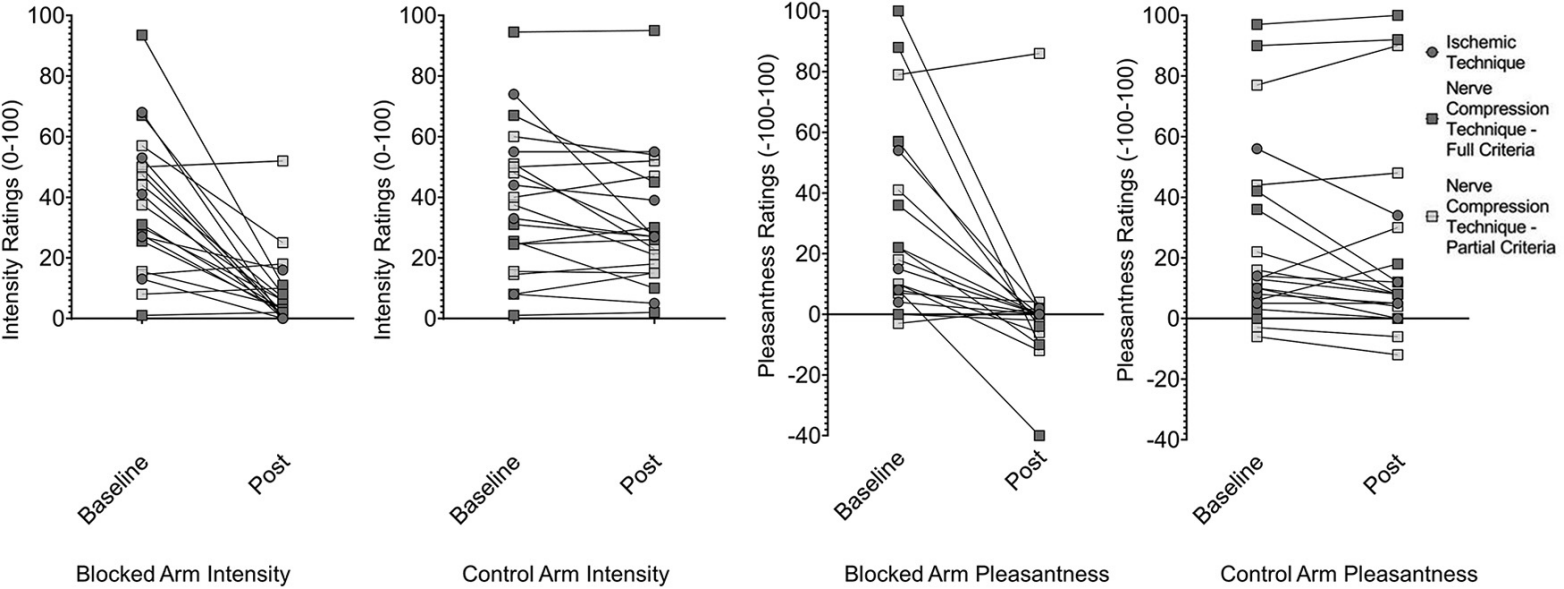
Effect of afferent A-fiber block on intensity and pleasantness of deep pressure. The intensity and pleasantness of deep pressure was rated after ischemic or compression nerve block, on sufficient loss of A-fiber associated sensation. Participants who met all preestablished criteria for nerve fiber separation and maintained the criteria after affective testing are labeled “full responders”; participants whose warmth perception rose >1°C or who did not maintain all criteria directly after the brushing task are labeled “partial responders.” For pleasantness ratings, negative numbers indicate unpleasantness.

The ischemic compression block successfully separated A- and C-fiber function in the five participants we report data from (of the two participants not analyzed here, one reported intolerable pain and one lost the ability to detect heat before vibration detection was affected). By around 20 min, vibration detection dropped from 100% to 0 in 4/5 participants, and 50% in the fifth, while heat detection thresholds remained unaffected (<1°C change in four subjects, <2°C in one). At that point in time, ratings of both intensity (previously reported by Case et al., 2021a) and pleasantness were nearly eliminated for both brushing (Fig. 3) and pressure (Fig. 4), but were largely unchanged in the control arm. Compared with baseline, nerve block reduced the pleasantness of both gentle brushing (blocked arm PRE M =* *43.6, SD =* *30.6, POST M =* *3.4, SD =* *6.5; control arm PRE M =* *43.8, SD =* *32.5, POST M =* *16.0, SD =* *14.6; *t*_(4)_ = 3.2, *p *=* *0.03, Cohen’s *d *=* *0.55; Table 1, line a) and deep pressure (blocked arm PRE M =* *19.2, SD =* *20.0, POST M =* *0.4, SD =* *0.9; control arm PRE M =* *18.6, SD =* *21.2, POST M =* *11.2, SD =* *13.4, trend; *t*_(4)_ = 2.2, *p *=* *0.09, Cohen’s *d *=* *0.91; Table 1, line b), as well as their intensity (gentle brushing blocked arm PRE M =* *24.8, SD =* *21.5, POST M =* *3.6, SD =* *4.6; control arm PRE M =* *27.8, SD =* *17.5, POST M =* *25.2, SD =* *19.0, trend, *t*_(4)_ = 2.1, *p *=* *0.1, Cohen’s *d *=* *1.6; Table 1, line c; deep pressure blocked arm PRE M =* *40.4, SD =* *21.5, POST M =* *3.2, SD =* *7.2; control arm PRE M =* *42.8, SD =* *24.7, POST M =* *30.6, SD =* *18.4, *t*_(4)_ = 3.3, *p *=* *0.03, Cohen’s *d *=* *1.7; Table 1, line d).

**Table 1:** Statistical table

	Data structure	Type of test	Power/Effect size
a	Non-normal	Linear mixed effects model	Cohen’s *d *=* *0.55
b	Non-normal	Linear mixed effects model	Cohen’s *d *=* *0.91
c	Non-normal	Linear mixed effects model	Cohen’s *d *=* *1.6
d	Non-normal	Linear mixed effects model	Cohen’s *d *=* *1.7
e	Non-normal	Linear mixed effects model	Cohen’s *d *=* *1.35
f	Non-normal	Linear mixed effects model	Cohen’s *d *=* *1.92
g	Non-normal	Linear mixed effects model	Cohen’s *d *=* *1.33
h	Non-normal	Linear mixed effects model	Cohen’s *d *=* *1.92
i	Non-normal	Pearson’s correlation	

### Study 2

The nerve compression block successfully separated A- and C-fiber function in 17 of the 24 study participants. Seven additional subjects were dismissed from their sessions (five reached the time limit without successful fiber separation, one reported intolerable pain, and one experienced abnormal nerve tingling before nerve block) and thus are not analyzed here. At ∼1 h (M =* *52.06 min), vibration detection dropped below 50% in all 17 of the analyzed participants (and was maintained after affective testing in 16/17 subjects). Cold detection thresholds dropped >5°C in all 17 subjects (and were maintained after affective testing in 15/17 subjects). At that time point, warmth detection thresholds remained within 1°C of baseline for 12 subjects, within 2°C for four subjects, and within 3°C for one subject (and were maintained at these levels in 15/17 subjects). Participants who met all preestablished criteria for nerve fiber separation and maintained the criteria after affective testing were labeled “full responders” (*N *=* *8) to the A-fiber nerve block; participants whose warmth perception rose >1°C or who did not maintain all criteria after affective testing were labeled “partial responders” (*N *=* *9).

At the time of maximal nerve fiber separation, the intensity and pleasantness of brushing were again nearly eliminated (Fig. 3), with significant reductions on the blocked arm relative to the control arm in both pleasantness (blocked arm PRE M =* *31.1, SD =* *34.0, POST M =* *5.8, SD =* *23.3; control arm PRE M =* *33.8, SD =* *3.3, POST M =* *31.1, SD =* *36.1, linear mixed effects model, *F*_(1,16)_ = 8.5, *p *=* *0.01, Cohen’s *d *= 1.35; Table 1, line e) and intensity (blocked arm PRE M =* *33.1, SD =* *25.0, POST M =* *5.3, SD =* *9.3; control arm PRE M =* *34.5, SD =* *24.5, POST M =* *32.8, SD =* *23.8, *F*_(1,16)_ = 22.2, *p* ≤ 0.001, Cohen’s *d *=* *1.92; Table 1, line f). Similarly, the intensity and pleasantness of deep pressure were also again nearly eliminated (see Fig. 4), with significant reductions on the blocked arm relative to the control arm in both pleasantness (blocked arm PRE M =* *31.0, SD =* *33.2, POST M =* *0.5, SD =* *25.1; control arm PRE M =* *28.8, SD = 33.1, POST M =* *25.5, SD =* *36.8, *F*_(1,15)_ = 10.6, *p *=* *0.005, Cohen’s *d *=* *1.33; Table 1, line g) and intensity (blocked arm PRE M =* *37.4, SD =* *23.6, POST M =* *10.0, SD =* *12.9; control arm PRE M =* *37.1, SD =* *24.2, POST M =* *31.8, SD =* *22.6, *F*_(1,15)_ = 17.8, *p* ≤ 0.001, Cohen’s *d *=* *1.92; Table 1, line h).

Across the two studies, changes in pleasantness ratings of brushing and pressure on the blocked arm were correlated, *r *=* *0.7, *N *=* *22, *p *<* *0.001. Changes in intensity ratings of brushing and pressure were similarly correlated, *r *=* *0.76, *N *=* *22, *p *<* *0.001 (Table 1, line i). Across the two studies, changes in pleasantness and intensity were not significantly correlated for either brushing (*r* = −0.15, *N *=* *22, *p* = 0.51) or pressure (*r *=* *0.30, *N *=* *22, *p *=* *0.17). However, on average, a similar magnitude of decrease was observed in pleasantness and intensity for both types of sensation (brushing intensity, M = −26.3, brushing pleasantness, M = −28.7, pressure intensity, M = −29.8, pressure pleasantness, M = −27.7).

## Discussion

The Social Touch Hypothesis (Vallbo et al., 1999; Morrison et al., 2010) proposes the dependence of the pleasantness of gentle skin stroking on C-tactile (CT) afferents, with additional contributions from afferent A-fibers and central processes (Vallbo et al., 2009). Recent findings, however, have suggested that afferent A-fibers alone might be sufficient in some cases to generate touch pleasantness (Marshall et al., 2019). In the present study, we conducted two type of nerve blocks in healthy adults to selectively reduce A- but not C-fiber function, in an attempt to determine the contribution of A-fibers to touch pleasantness of CT-targeted gentle brushing, as well as deep pressure. Our findings demonstrate that after loss of A-fiber sensation, the perceived intensity and pleasantness of gentle brushing and deep pressure are nearly abolished, and these ratings changes are highly correlated. In contrast, these perceptions are maintained in the control arm. These novel findings strongly suggest that afferent A-fiber input- presumably Aβ mechanoreceptive A-fiber- is necessary in the moment of touch for explicit ratings of touch pleasantness, in healthy adults.

In Study 1, a near complete loss of both intensity and pleasantness of gentle brushing and deep pressure was observed after ischemic nerve blockade. This method of nerve block is highly efficient at separating A- and C-fiber function, and blocks somatosensory innervation of the full lower arm. However, it causes a significant amount of discomfort and pain, leaving questions about the effect of this pain on ratings of touch pleasantness. To address this limitation, Study 2 conducted a very similar design using a nerve compression block. This block takes longer to take effect (∼1 h) and affects a much smaller region of skin (dorsal hand near thumb and forefinger)- but does so with minimal discomfort or pain. Study 2 obtained a nearly identical result: near complete loss of both intensity and pleasantness of gentle brushing and deep pressure after the nerve block. In both studies, touch pleasantness and intensity were maintained on the control arm, suggesting that results cannot be attributed to effects of the nerve block procedure on mood, or distracting effects of pain and discomfort. These techniques provide convergent evidence for the dependence of explicit touch pleasantness ratings on afferent A-fibers.

The importance of mechanoreceptive A-fibers to touch pleasantness is consistent with a growing recognition of the complexity of afferent processes in the spinal cord (Abraira et al., 2017; Marshall et al., 2019) and brain (Hagberg et al., 2019; Schirmer et al., 2022), as well as the role of central processes (McCabe et al., 2008; Vallbo et al., 2009; Ellingsen et al., 2016; Fotopoulou et al., 2022), in touch pleasantness. It is also consistent with the pleasantness of touch on the glabrous skin of the hand, although the contribution of its sparse CT innervation is not clear (Löken et al., 2011; Klöcker et al., 2012; Cruciani et al., 2021).

Our results are additionally in line with the findings of Marshall and colleagues (Marshall et al., 2019; Marshall and McGlone, 2020), who reported that ablation of the Lamina I-anterolateral pathway at C1/C2 reduced perception of pain, temperature, and itch, but not the pleasantness of slow stroking (Marshall et al., 2019). The Lamina I-anterolateral pathway is the putative spinal pathway for unmyelinated afferents projecting to the thalamus, as well as the spinohypothalamic and spinoparabrachial pathways. Their result suggests the sufficiency of the dorsal column pathway for explicit perception of touch pleasantness. This could be because of CT fibers joining or modulating the dorsal column pathway below the level of ablation- Marshall and colleagues’ “alternate pathway hypothesis” (Marshall and McGlone, 2020). Our data confirm a critical role of A-fibers, likely Aβ mechanoreceptive afferents. Our data are less clear regarding Marshall and colleagues’ “alternate percept hypothesis,” in which early social touch experiences condition associations between A- and C-fiber signals, explaining the sufficiency of dorsal column input. We propose a modified “alternate percept hypothesis” in which C-fibers condition responses to affective touch, but cannot be interpreted in the absence of corresponding A-fiber input.

Our results additionally demonstrate that afferent A-fibers are critical for the interpretation of the pleasantness of deep pressure. This is not surprising, given the aforementioned association of deep pressure sensation with innervation of deeper tissues suggested by multiple animal and human studies (Kellgren and McGowan, 1948; Kaufman et al., 1983; Mense and Meyer, 1985; Abrahams, 1986; Lewin and McMahon, 1991; Graven-Nielsen et al., 2004), as well as our work demonstrating its non-*Piezo2* mechanism, which differs from light touch sensation (Case et al., 2021a). However, the potential contributions of CT fibers to deep pressure pleasantness are unknown.

Our findings are limited by the fact that it is not possible to fully separate A- and C-fiber function by means of nerve block. To mitigate this challenge, we have performed two methods of nerve block whose strengths and limitations complement one another. Through this approach we provide strong convergent evidence for the reliance of gentle stroking pleasantness on A-fiber afferents. An additional limitation to our data is that participants cannot be fully blinded to the nerve block procedure; sensory changes are self-evident. However, participants were naive to the timeline of anticipated sensory effects and were told that effects of the nerve block on many forms touch are unknown. While we demonstrate that explicit touch pleasantness ratings are highly impacted by A-fiber nerve block, it remains to be tested whether implicit measures of affective response are similarly impacted, confirming the dependence of the full range of CT affective effects on the contribution of afferent A-fibers. For example, CT-targeted touch preferentially activates the zygomaticus “smiling” muscle (Pawling et al., 2017) and increases heart rate variability (Triscoli et al., 2017). Finally, follow-up work is needed to test the mechanisms for a greater variety of affective touch stimuli, including pressure of varying levels, frequencies, and locations.

In sum, our data from two nerve block techniques performed to block afferent A-fiber input in healthy adults confirms that in healthy adults, at the moment of touch, both A- and C-fiber afferents are important contributors to the pleasantness of CT-targeted gentle stroking and deep pressure. This study expands our understanding of the somatosensory pathways that underlie the affective and social effects of touch, and may inform future targets for noninvasive modulation of affect.

## Synthesis

Reviewing Editor: Erica Glasper, The Ohio State University Decisions are customarily a result of the Reviewing Editor and the peer reviewers coming together and discussing their recommendations until a consensus is reached. When revisions are invited, a fact-based synthesis statement explaining their decision and outlining what is needed to prepare a revision will be listed below. The following reviewer(s) agreed to reveal their identity: NONE.

Synthesis: In this study, the authors used peripheral nerve blocks to understand the role of A-fibers in touch perceptions. This is a worthy area of exploration, as it highlights the necessity of afferent A-fibers for conscious touch perception. Importantly, these findings indicate that tactile pleasantness includes more than C-tactile (CT) firing and likely involve the sub-conscious aspects of gentle touch only. This work advances our understanding of the complexities of touch processing. The manuscript is well-written, and findings will advance the field. There are, however, issues with interpretation of the existing literature as it pertains to CT afferents and pleasant touch. The current view appears too simplistic. When appropriate, “direct quotes” from the reviewers are included within the comments below to increase clarity. A revision of the work is welcomed.

Major Comments

1. Overall

a. The authors should reconsider the existing literature’s role in postulating that pleasant touch involves more than CT firing. Doing so does not reduce the novelty of the current work. The authors are encouraged to highlight the need for many different afferent inputs, demonstrating pleasantness is a complex central process.

i. The thought that pleasant touch requires A-beta mechanoreceptor input is not a new thought, but it appears to have been ignored. This theory was postulated at the formulation of the affective touch hypothesis (Vallbo et al, 2009, Encyclopedia of Neuroscience, chapter ‘Pleasant touch’). This hypothesis suggests that CT afferents are directly involved in pleasant touch but involve the necessity of other afferent and central processes. It has been postulated that CTs play a conditioning/reinforcement role (e.g., summarized in Ackerley 2022, Curr Op Beh Sci; Croy et al, 2022 Curr Op BehSci).

ii. Many studies have demonstrated the importance of conscious touch signals in pleasant touch (e.g., sensing/assessing pleasantness using the glabrous hand skin, Löken et al, 2011 Brain Res; Klöcker et al, 2012, Front Neurorobot; Cruciani et al, 2021 Neurosci Biobeh Rev). This is further demonstrated in studies of deafferented patients that lack conscious touch perception - these patients cannot rate pleasantness like typical participants. Additionally, the “study by Morrison et al on patients with reduced C-fibers also shows that although ratings may be a little lower, they can clearly evaluate tactile pleasantness, with few CTs”.

b. The authors should clarify what is meant by ‘A-fibers’. “In physiological terms, A-fiber could refer to motor efferents or all types of afferent A-fiber (proprioceptive, mechanoreceptive, thermoreceptive, nociceptive).”

2. Methods

a. Please add more details to the methods.

i. Line 129 - Was the oscillating deep pressure provided to the entire hand? What was the make/model of the hand massager and what type of stimulation was provided “(e.g., frequency of oscillation, force/strength of pressure)?

ii. Study 1 - Please state what hand received the stimuli. Did this vary depending on handedness in the participants?

iii. Did any of the same participants participate in both Study 1 and Study 2?

iv. Please provide more details regarding the application of deep pressure in Study 2 using the handheld rolling massage ball. What area was stimulated? How was the ball applied to the skin?

3. Results

a. The authors should consider making direct comparisons (i.e., difference computations) between pleasantness and the intensity rankings for stroking and pressure.

4. Figures

a. To increase readability of Figures 3 and 4, the authors should add pleasantness and intensity to the titles or labels.

b. Please clarify the pleasantness ratings in Figures 3 and 4. Specifically, did the range only go from 0 to 100? Was there never an unpleasant sensation resulting in a negative number?

5. Discussion

a. The argument in the discussion should be revised to include existing literature suggesting that other mechanisms may contribute to the perception of pleasant touch.

b. “In the discussion, especially the middle of p. 10, the authors should accept that many studies already show that conscious tactile pleasantness likely comes A-beta mechanoreceptors, from studies on pleasantness of touch on the glabrous skin (with no or few CTs) to the work on patients (as outlined above).”

c. Lines 423-427 - Please clarify the relationship between CT afferents and facial expression. Are the authors suggesting that the facial expression directly relates to pleasant touch?

d. Please increase discussion of the limitations and implications of this work. For example, the present work focused on the hand, but deep pressure perception may be different throughout the body. Also, could the method of pressure be relevant (e.g., moving across the body or stationary)? The authors may also consider the “link between pleasantness and itch, see the study of Mochizuki et al (2017, Itch)”.

Minor Comments

1. Introduction

a. Line 55 - Please clarify this statement: ‘A-fiber deafferentation show preserved CT touch pleasantness’. Do the authors mean to imply that these patients have a “conscious perception of tactile pleasantness”?

b. Lines 72-74 - Please clarify what is meant by ‘the causal role of A versus CT inputs in the pleasantness of gentle stroking. However, this causal has never been directly tested in healthy humans’. It is unclear if the authors are implying that CT cause pleasantness.

2. Results

a. The description of the nerve block in the results was appreciated.

b. Line 280 - Please clarify what is meant by ‘A- and - fiber function’.

c. Line 329 (and elsewhere) - Please change ‘0.0007’ to <0.001 when very small significance levels are needed.

d. What do the random letters after the Cohen’s d values in Study 2 represent?

3. Discussion

a. 3rd paragraph - Please include the MEG paper authored by Hagberg et al, 2019 (NeuroImage) along with the EEG paper that is already cited.

4. Figures

a. 1c - Please clarify whether the vibration was on the dorsal hand (like in Study 2) or whether it occurred on the dorsal forearm.
